# Imaging and cancer survivorship: challenges and changing concepts

**DOI:** 10.1186/1470-7330-15-S1-O33

**Published:** 2015-10-02

**Authors:** Kenneth A Miles, Dalveer Singh

**Affiliations:** 1 Department of Diagnostic Imaging, Princess Alexandra Hospital, Woolloongabba, Queensland, 4102, Australia; 2 Institute of Nuclear Medicine, University College London, London, NW1 2BU, UK

## 

The widening gap between cancer incidence and mortality testifies to the increasing success of cancer treatment. For example, in Queensland Australia, there are now more than 7 cancer survivors for each patient newly diagnosed with cancer. These individuals represent a population at risk for recurrent malignancy for whom there is a growing demand for imaging services.

Imaging for suspected recurrence may be prompted by symptoms or rising tumour markers, whilst for some tumours, imaging contributes to regular surveillance. The benefits of early detection of recurrence through imaging surveillance need to be balanced against cost, patient anxiety and radiation exposure. A recent study has estimated the absolute risk of second cancer induction resulting from use of radiological examinations in this context to be between 0.1% and 10% [1]. Targeting patients at greatest risk of recurrence would improve the balance between risk and benefit for imaging surveillance and is potentially achievable through the use of prognostic imaging biomarkers derived from imaging procedures performed at staging or post-treatment [2].

Increasingly sophisticated treatments for recurrent disease present further challenges to the use of imaging in cancer survivorship. There are now more therapeutic options for patients with localised disease or recurrence to a few sites (oligometastatic disease). Although well established for hepatic metastases from colorectal cancer, surgical resection of localised recurrence is increasingly adopted for alternative sites of recurrence and for other tumour types. For tumour sites unamenable to surgery, stereotactic body radiotherapy can allow focal delivery of high-dose radiation with single or few fractions with promising local control and overall survival rates [3].

These developments create new questions for imaging. Firstly, should imaging surveillance programmes of asymptomatic cancer survivors be developed to allow identification of the oligometastatic state prior to disseminated disease? The potential benefits of surveillance are highlighted by a study of patterns of distant failure and progression in breast cancer which found oligometastatic disease to be more common amongst asymptomatic patients [4]. Secondly, how can imaging be optimised to distinguish oligometastatic from disseminated disease? Imaging in this context must have high sensitivity and specificity on a lesion-by-lesion basis because an accurate assessment of the number of metastases is required to avoid the morbidity of inappropriate oligometastatic treatment, as well as the possibility of oligometastatic treatment being wrongly withheld due to the presence of benign lesions that are indistinguishable from additional metastases. Hybrid imaging techniques that combine modalities with complementary sensitivity and specificity are likely to offer the greatest opportunities for accurate assessment of oligometastatic disease. Higher accuracy for skeletal metastases can be achieved by diphosphonate SPECT/CT or fluoride PET/CT in place of planar scintigraphy [5] whilst integrated FDG-PET/MRI has the potential to optimise detection of skeletal and hepatic recurrence in a single examination [6,7]. The new PET tracer Gallium-68 Prostate Specific Membrane Antigen demonstrates high accuracy for nodal and skeletal recurrence prostate cancer [8].

Imaging protocols for cancer staging are now well established. Increasing cancer survivorship has created a need to develop equivalent protocols for the diagnosis and assessment of tumour recurrence.
